# Mesoscale Simulation Based on the Dynamic Mean-Field Density Functional Method on Block-Copolymeric Ionomers for Polymer Electrolyte Membranes

**DOI:** 10.3390/membranes13030258

**Published:** 2023-02-22

**Authors:** Hoseong Kang, Muyeong Cheon, Chang Hyun Lee, Tae-Ho Kim, Young Taik Hong, Sang Yong Nam, Chi Hoon Park

**Affiliations:** 1 Department of Energy Engineering, Future Convergence Technology Research Institute, Gyeongsang National University (GNU), Jinju 52725, Republic of Korea; 2 Energy Engineering Department, College of Engineering, Dankook University, Cheonan 31116, Republic of Korea; 3 Center for Membranes, Korea Research Institute of Chemical Technology (KRICT), Daejeon 34114, Republic of Korea; 4 Department of Materials Engineering and Convergence Technology, Gyeongsang National University (GNU), Jinju 52725, Republic of Korea

**Keywords:** block copolymers, proton exchange membranes (PEMs), mesoscale simulation, phase separation, water channel

## Abstract

Block copolymers generally have peculiar morphological characteristics, such as strong phase separation. They have been actively applied to polymer electrolyte membranes for proton exchange membrane fuel cells (PEMFCs) to obtain well-defined hydrophilic regions and water channels as a proton pathway. Although molecular simulation tools are advantageous to investigate the mechanism of water channel formation based on the chemical structure and property relationships, classical molecular dynamics simulation has limitations regarding the model size and time scale, and these issues need to be addressed. In this study, we investigated the morphology of sulfonated block copolymers synthesized for PEM applications using a mesoscale simulation based on the dynamic mean-field density functional method, widely applied to investigate macroscopic systems such as polymer blends, micelles, and multi-block/grafting copolymers. Despite the similar solubility parameters of the monomers in our block-copolymer models, very different morphologies in our 3D mesoscale models were obtained. The model with sulfonated monomers, in which the number of sulfonic acid groups is twice that of the other model, showed better phase separation and water channel formation, despite the short length of its hydrophilic block. In conclusion, this unexpected behavior indicates that the role of water molecules is important in making PEM mesoscale models well-equilibrated in the mesoscale simulation, which results in the strong phase separation between hydrophilic and hydrophobic regions and the ensuing well-defined water channel. PEM synthesis supports the conclusion that using the sulfonated monomers with a high sulfonation degree (32.5 mS/cm) will be more effective than using the long hydrophilic block with a low sulfonation degree (25.2 mS/cm).

## 1. Introduction

Block copolymers consisting of two or more different oligomers generally have peculiar morphological characteristics such as interpenetration, self-assembly, and strong phase separation depending on the property of each block, and they have been widely used for various industrial applications [1,2]. Among them, one of the active research areas is the use of block copolymers for proton exchange membranes (PEMs) for fuel cell applications [3,4,5,6,7]. Proton exchange membrane fuel cells (PEMFCs) using hydrogen and oxygen as fuels can generate electrical energy through electrochemical oxidation–reduction reactions. Accordingly, the performance of PEMFCs depends on how effectively protons are transferred through the PEM as an electrolyte [8,9,10,11].

In the fuel cell types operated under low- to medium-temperature conditions, PEMs absorb water molecules so that the water channel is formed inside the hydrated PEMs, and protons are transferred along the water channel [9,10,12]. So far, to address this issue, many researchers have designed the water channel morphologies to have broader and more connected proton pathways in synthetic approaches such as block-copolymer PEMs, high-free volume PEMs, grafted/branched copolymer PEMs, and highly sulfonated monomer-based PEMs. It is also applied in physicochemical tuning approaches such as crosslinking, surface fluorination, thermal annealing, and organic–inorganic nanocomposites [10]. Among these approaches, focusing on the self-assembly and phase separation mentioned above as representative morphological characteristics of block copolymers, there has been extensive research on novel block-copolymer PEM architectures consisting of the hydrophobic domain mechanically supporting PEMs and the hydrophilic domain forming water channels inside. This has demonstrated better fuel cell performance, such as proton conductivity and electrical power generation, than conventional homo- or random-copolymer PEMs [3,4,5,6,7,11].

Accordingly, to correlate the PEM architecture with fuel cell performance, various studies have been performed to characterize the morphology of the hydrophilic domains/water channels inside PEMs and the proton transport behavior [11,12,13]. However, there is a limit to fully understanding the water channel morphology and phase separation phenomena in the PEMs under the actual hydration conditions by only using experimental characterization. Therefore, a molecular simulation method based on computational chemistry capable of understanding materials at the atomic and molecular levels has been actively studied to identify water channel formation and phase separation phenomena in the PEMs [14,15,16,17,18]. Mesoscale simulation is a well-known molecular simulation technique that can simulate a larger model for a more extended period than molecular dynamics (MD); the former calculates the group of atoms as a single unit called a bead, while the latter calculates an atom as a minimum unit [14,15,19,20,21,22,23,24,25]. In addition, unlike the computational simulation of finite element analysis using fluid dynamics and numerical methods that do not calculate atoms and molecules, a mesoscale simulation technique can demonstrate the distribution, movement, and morphology of atoms and molecules composed of the atoms expressed through beads [14,19,20,21,22,23,24,26,27]. As a result, mesoscale simulations have been widely applied to investigate macroscopic systems such as polymer blends, micelles, and multi-block/grafting copolymers [14,19,20,21,22,23,24].

In this study, the ability of the mesoscale simulation technique to help us understand the water channel morphology and phase separation phenomenon in the block-copolymer PEMs under the actual hydration conditions was investigated. Two block copolymers with similar chemical structures except for sulfonated monomers were chosen [28]. Using mesoscale simulation, we tried to demonstrate how the difference in the chemical structures affects the macroscopic properties. Finally, the simulated results were compared to the experimental ones to confirm the usefulness of mesoscale simulations and to suggest the effective chemical structure for high proton conductivity, focusing on the location and the number of sulfonic acid groups, which are still under debate.

## 2. Materials and Methods

### 2.1. MD Simulation for Solubility Parameter Calculation

The solubility parameters used to obtain the study’s interaction parameters for mesoscale simulation were calculated using molecular dynamics (MD). The solubility parameters are computed from the cohesive energy per unit volume (CED) as follows:

(1) $$\delta =[CED{]}^{\frac{1}{2}}={\left[\frac{\Delta H-RT}{V_{m}}\right]}^{1/2}$$

where *δ, R, T, H*, and *V_m_* indicate the solubility parameter, the gas constant, the temperature, the heat of vaporization, and the molar volume, respectively [29,30,31]. Our models’ CED and solubility parameters were obtained using the Forcite module in the Materials Studio program package (Biovia Inc., San Diego, CA, USA).

The Flory–Huggins interaction parameter (χ) was calculated as the interaction parameter between two beads, *i* and *j*, from their solubility parameters (δ) [32,33,34,35]:

(2) $$\chi =\frac{V_{ref}{\left(\delta_{i}-\delta_{j}\right)}^{2}}{RT}$$

where *V_ref_* indicates the reference volume (i.e., the mean molar volume of the two monomers, *i* and *j*), *R* denotes the gas constant, and *T* indicates the temperature [32,33].

First, the sulfonated and non-sulfonated monomers were chosen as the representative beads of hydrophilic and hydrophobic domains, respectively. After geometry optimization of the monomers, they were polymerized with 50 repeating units of each monomer, and the resulting polymer backbones were geometrically optimized again. In this study, COMPASS II (Condensed-phase Optimized Molecular Potentials for Atomistic Simulation Studies II) [36,37,38] was used as a force-field (Equation (3)), and the force-field type and charge value for each atom were set as defaults, which were verified from the results of the previous studies [39]. Using these polymer backbone structures, hydrophilic sulfonated-polymer 3D models and hydrophobic polymer 3D models were generated using the Amorphous Cell module. Then, the structure of each 3D model’s geometry was optimized by the Forcite module. Here, the smart algorithm using a cascade of the steepest descent, ABNR, and quasi-Newton methods was applied by setting the convergence tolerance to 0.001 kcal/mol for the maximum energy change and 0.5 kcal/molÅ for the leading force.

(3) $$\begin{array}{ll} E_{pot}=\sum_{b}[K_{2}(b & {-b_{0})}^{2}+K_{3}{\left(b-b_{0}\right)}^{3}+K_{4}{\left(b-b_{0}\right)}^{4}]+\sum_{\theta }[H_{2}{\left(\theta -\theta_{0}\right)}^{2}+H_{3}{\left(\theta -\theta_{0}\right)}^{3}+H_{4}{\left(\theta -\theta_{0}\right)}^{4}\\ & +\sum_{\phi }\left[V_{1}[1-\cos(\phi )]+V_{2}[1-\cos(2\phi )]+V_{3}[1-\cos(3\phi )]\right]+\sum_{x}K_{x}x^{2}\\ & +\sum_{b}\sum_{b^{\prime }}F_{bb^{\prime }}\left(b-b_{0}\right)\left(b^{\prime }-b_{0}^{\prime }\right)+\sum_{\theta }\sum_{\theta^{\prime }}F_{\theta \theta^{\prime }}\left(\theta -\theta_{0}\right)\left(\theta^{\prime }-\theta_{0}^{\prime }\right)\\ & +\sum_{b}\sum_{\theta }F_{b\theta }\left(b-b_{0}\right)\left(\theta -\theta_{0}\right)+\sum_{b}\sum_{\phi }\left(b-b_{0}\right)\left(V_{1}\cos\phi +V_{2}\cos2\phi +V_{3}\cos3\phi \right)\\ & +\sum_{b^{\prime }}\sum_{\phi }\left(b^{\prime }-b_{0}^{\prime }\right)\left(V_{1}\cos\phi +V_{2}\cos2\phi +V_{3}\cos3\phi \right)\\ & +\sum_{\theta }\sum_{\phi }\left(\theta -\theta_{0}\right)(V_{1}\cos\phi +V_{2}\cos\phi +V_{3}\cos\phi )+\sum_{\phi }\sum_{\theta }\sum_{\theta^{\prime }}K_{\phi \theta \theta^{\prime }}\cos\phi \left(\theta -\theta_{0}\right)\left(\theta^{\prime }-\theta_{0}^{\prime }\right)\\ & +\sum_{i>j}\frac{q_{i}q_{j}}{\varepsilon_{r_{ij}}}+\sum_{i>j}\left[\frac{A_{ij}}{r_{ij}^{9}}-\frac{B_{ij}}{r_{ij}^{6}}\right]\\ & +\sum_{i>j}\left\{D_{0}\left[{\left\{\exp\left(-\left(\frac{𝒴}{2}\right)\left(\frac{r_{ij}}{R_{0}}\right)\right)\right\}}^{2}-2\exp\left(-\left(\frac{𝒴}{2}\right)\left(\frac{r_{ij}}{R_{0}}-1\right)\right)\right]f_{s}-\left(1-f_{s}\right)\frac{C_{6}}{r_{ij}^{6}}\right\} \end{array}$$

Next, the equilibration step was performed by combining MD calculations: (1) NPT (constant number of atoms, pressure, and temperature) quench calculation at 298 K for 50 ps and then 698 K for 50 ps under 1 atm; (2) NPT MD calculation at 298 K for 50 ps under 1 atm; (3) NPT MD calculation at 298 K for 50 ps under 1 GPa; (4) NVT MD calculation at 698 K for 20 ps; (5) NVT MD calculation at 298 K for 20 ps; and (6) NPT MD calculation at 298 K for 100 ps under 1 atm. In the equilibration step, steps (3) to (6) were repeated until the density change of the 3D models was stabilized within the range of 1%. Here, the quench calculation employing the Forcite module was used because it is difficult to obtain an optimized structure of polymer models with rigid aromatic chains only by means of a simple-structure optimization tool due to the characteristics of a long main chain and various twisted forms. Therefore, a thermal condition is given to a polymer 3D model to induce thermal movement of the main chain in the quenching process. Their optimized structures were obtained by sampling the thermally ‘excited’ 3D models with various conformations and configurations and then geometrically optimizing them [12,23]. In the equilibration steps (3) to (6), the external pressure causes the main chains to become close to each other and overcome the chain rigidity, which can fix the abnormal cavity inside the polymer 3D models with stiff and rigid main chains. In addition, the high simulation temperature, generally over the glass transition temperature of a target polymer, can provide excessive mobility to the atoms and then also fix the abnormal cavity [12,19,39].

Finally, solubility parameter calculations were performed using the Forcite module. In addition, the solubility parameters of our models were calculated using the Synthia module in the Materials Studio program package (Biovia Inc. San Diego, CA, USA), which is based on the quantitative structure–property relationship (QSPR) methods [23,33], and we compared them to the results from MD calculation.

### 2.2. Mesoscale Simulation

Unlike MD, which directly calculates each atom in a model system, mesoscale simulations estimate each bead, a group of atoms representing similar characteristics [40,41,42]. Therefore, the computation time can be significantly reduced compared with the molecular dynamics, so that the object’s size can be simulated and the simulation time scale can increase [14]. Recently, based on the iterative Boltzmann inversion method and the reverse mapping method, the mesoscale simulation strategy combining the coarse-grained simulation and the atomistic simulations showed good agreement with the experimental data in the polyelectrolyte field [43,44,45,46,47]. However, there are very limited research groups reporting those mesoscale simulation results due to high technological barriers and programming techniques. Accordingly, the classical mesoscale simulation technique was adopted in this study so that the experimentalist could easily perform the mesoscale simulation for their materials. As mentioned in Section 2.1, for the mesoscale simulation, monomers with no sulfonic acid groups and monomers with sulfonic acid groups, constituting the structure of our sulfonated polymer models, were assigned as hydrophobic and hydrophilic beads, respectively. Mesoscale polymer models were designed based on the molecular weight of the polymer models and the hydrophilic/hydrophobic monomer ratio from the experimental data, which will be discussed in Section 3.1. A 3D model of the hydrated sulfonated polymer was used to express a mesoscale polymer bead model, and a water bead model was inserted according to the volume ratio calculated from the experimental water intake and bead volume. The mesoscale simulation was performed using the MesoDyn module in the Materials Studio program package (Biovia Inc., USA). MesoDyn is based on mean-field density functional theory (DFT), in which the fluid or fluid-like materials are described by the concentration fields of the various components in the system [23,38]. The evolution of the concentration field *ρ_A_*(*r*) and the change in the potential external *U_A_*(*r*) are correlated by the derivative of the partition function,

(4) $$\rho_{A}\left(r\right)=n_{A}kT\frac{\partial \ln\phi }{\partial U_{A}\left(r\right)}$$

where *k, T, n_A_*, and *ϕ* indicate the Boltzmann constant, the temperature, the number of chains, and the intramolecular partition function, respectively. The chains instantaneously equilibrate in this process, and the free energy is minimized. The free energy function can be written as follows [23,34]:

(5) $$F [\left\{\rho_{A}\right\}]=-kT\sum_{i}\ln\frac{\phi_{i}^{n_{i}}}{N_{i}!}-\sum_{i}\int_{V}U_{A}\left(r\right)\rho_{A}\left(r\right)dr\;+\frac{1}{2}\underset{A,B}{\sum }\iint_{V^{2}}\varepsilon_{AB}\left(\left|r-r^{\prime }\right|\right)\rho_{A}\left(r\right)\rho_{B}\left(r^{\prime }\right)drdr^{\prime }\;+\frac{\kappa_{H}}{2}\int_{V}{\left(\underset{A}{\sum }\upsilon_{A}\left(\rho_{A}\left(r\right)-\rho_{A}^{0}\right)\right)}^{2}dr$$

where *V*, $\rho_{A}^{0}$ , and *κ_H_* indicate the system volume, the average density of each field with bead volume νA, and the Helfand compressibility parameter, respectively. The first two terms constitute the ideal free energy, and the third indicates the effect of the interactions between chains (Equation (6)).

(6) $$\varepsilon_{AB}\left(\left|r-r^{\prime }\right|\right)=\varepsilon_{AB}^{0}{\left(\frac{3}{2\pi a^{2}}\right)}^{\frac{3}{2}}exp [-\frac{3}{2a^{2}}{\left(r-r^{\prime }\right)}^{2}]$$

where *a* indicates the Gaussian bond length. The interaction energies $\varepsilon_{IJ}^{0}$ are related to the Flory–Huggins χ parameter as follows:

(7) $$\nu^{-1}\varepsilon_{AB}^{0}=\chi_{AB}RT$$

The last term in Equation (5) accounts for the compressibility of the system by controlling the density fluctuation.

These potential These fields evolve dynamically due to random “thermal” noise but also because of gradients in the chemical potential via stochastic diffusion of the density fields (Equation (8)). chemical differences arise because of asymmetric interactions between the various species.

(8) $$\frac{\partial \rho_{A}}{\partial t}=M\nu \nabla \cdot \rho_{A}\rho_{B}\nabla [\mu_{A}-\mu_{B}]+\eta \;\frac{\partial \rho_{B}}{\partial t}=M\nu \nabla \cdot \rho_{A}\rho_{B}\nabla [\mu_{B}-\mu_{A}]+\eta$$

where *M, Μa*, and *η* indicate the bead mobility parameter, the chemical potential, and the Gaussian noise distribution, respectively.

As the time step was set to 50 ns and the number of steps was 1000 and 10,000 for each mesoscale model, the final 3D models were obtained with total simulation times of 50 and 500 μs in this study. The total grid dimensions were 32 nm × 32 nm × 32 nm, with a grid spacing parameter of 1.0 nm at 298 K. Since the average bead-diffusion coefficient was set to 1.0 × 10^−7^ cm^2^/s, the dimensionless time step of 0.5, as the product of the time step and the bead diffusion coefficient, divided by the square of the grid spacing [23,38], was used in the MesoDyn simulation. The solve space for the DFT solver was chosen to be mixed (density and potential), which is the traditional method of performing dynamics calculations in MesoDyn, in which the calculation is carried out by cycling between density and potential space [23,38]. The solver tolerance was set to 0.001 and the maximum iterations to 100 per step.

## 3. Results and Discussion

### 3.1. Sulfonated Polymer Models and Their Solubility Parameters

**Figure 1:**
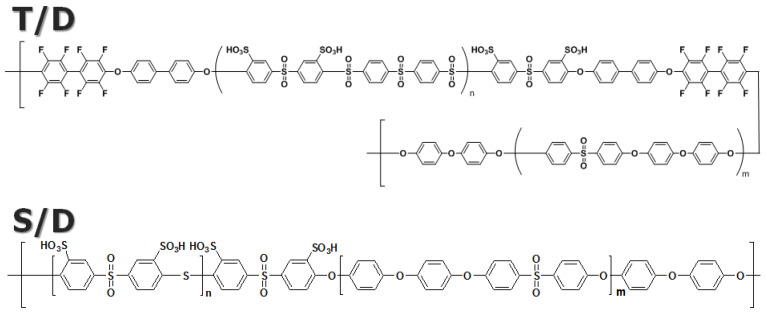
Chemical structures of sulfonated polyarylene sulfone-multiblock copolymers used as PEM models in the mesoscale simulation of this study.

Figure 1 shows the chemical structures of sulfonated polyarylene sulfone-multiblock copolymers used as PEM models in the mesoscale simulation of this study. Our models display significant differences in the characteristics of the chemical structures compared to the well-known commercial PEM, Nafion [10]: (1) focusing on the polymer backbone structure, the former are categorized as non-perfluorinated (or sulfonated hydrocarbon) PEMs, but the latter are categorized as perfluorinated PEMs; and (2) focusing on the location of sulfonic acid groups, the functional groups are directly introduced into the backbone in the former, but those are attached to the end of the side chain in the latter. These differences significantly affect their phase separation and water channel formation [10,12,39], which will be discussed in Section 3.2.

As shown in Figure 1, our models, designated as TD and SD, are polyarylene sulfone-type polymers and thus show a similar structure. In the TD model, the sulfonated monomer and the non-sulfonated monomer are designated as T and D, respectively. The chemical structure of the hydrophobic block of the SD model is the same as the non-sulfonated D monomer of TD. However, the hydrophilic block shows a different chemical structure from TD, where the sulfonated S monomer has a higher degree of sulfonation than the sulfonated D monomer. This difference could be greater in terms of the whole chemical structure of the polymer chains. Still, the characteristics of the actual PEMs prepared using these polymer structures show a significant difference, as shown in Table 1. Mainly, there is a substantial difference in the water uptake, which is the most important factor in PEMs for fuel cells.

Table 2 shows the solubility parameters predicted by the QSPR method and calculated by the MD simulation and PEM model information in this study. The values from the Synthia module are more significant than those from the MD simulations. The solubility parameter of the non-sulfonated monomer, D, shows a more substantial deviation than those of the sulfonated monomers, T and S, which is as much as the deviation between methanol and dimethyl sulfoxide (DMSO). Accordingly, the solubility parameter should be carefully chosen in the mesoscale simulation. In the case of water beads, W, the solubility parameter of 25 MPa^1/2^ reported in the literature was used [23,39]. PEM models were built based on the molecular weights experimentally measured, as shown in Table 2; due to the small length of the S bead, two monomers were mapped into one bead. In this study, PEMs with similar molecular weights of hydrophilic and hydrophobic blocks were selected in each block-copolymer structure to observe the difference in phase separation and water channel formation. However, in the case of the SD model, since the number of sulfonic acid groups in the S monomer is twice that of the sulfonic acid groups in the T monomer, the IEC value is much higher than that of the TD model (Table 1). Accordingly, it is observed that the difference in the IEC affects a difference in water uptake, and consequently, the water uptake of SD is very large as compared with TD.

### 3.2. Mesoscale Simulation

**Figure 2:**
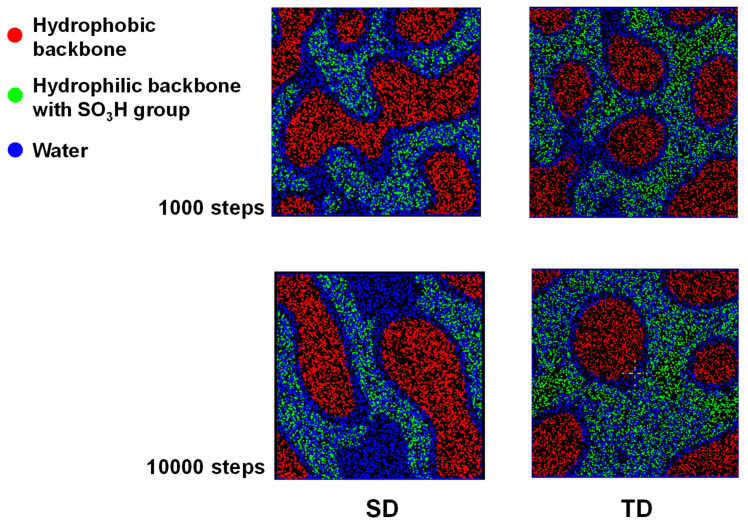
Sliced images of 3D mesoscale results at 298 K after 1000 and 10,000 steps.

Figure 2 shows the sliced images of 3D mesoscale results at 298 K after 1000 and 10,000 steps. Compared with the PEMs in our previous results [48], large differences can be observed between SD and TD models. Neither model can show distinct phase separation as much as Nafion does. However, the SD model has a much stronger phase separation between hydrophilic and hydrophobic regions than the TD model and forms a more effective water channel than our previous PEM with a similar IEC (2.41 meq/g). On the contrary, the TD model has a very similar sliced image to our previous PEM model, displaying a similar IEC (1.82 meq/g). In particular, the water channel in the SD model can be observed even after a relatively short 1000 steps.

**Figure 3:**
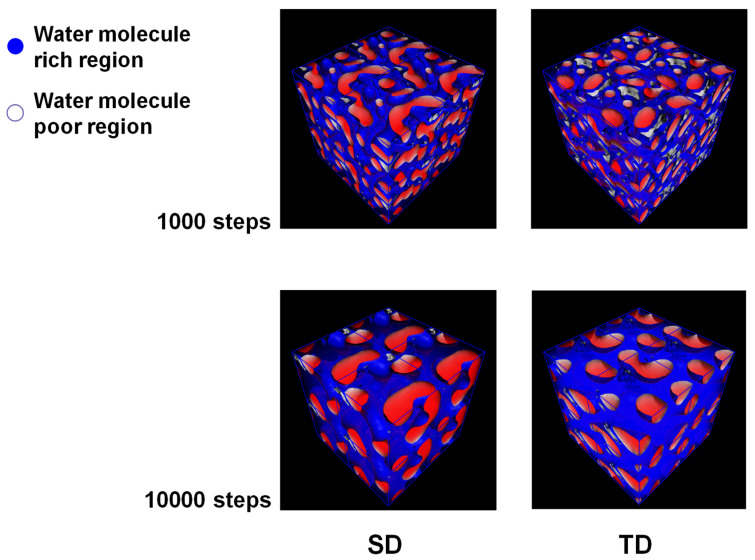
Three-dimensional images of the mesoscale results at 298 K after 1000 and 10,000 steps.

Since water beads and sulfonated monomer beads are distributed together in Figure 2, to observe the water channel more clearly, only the water bead-rich and -poor regions are shown in Figure 3 after removing the sulfonated monomer beads. Here, the water-rich part is located on the blue side of the isosurface. The area in which the water molecules are relatively sparse is located on the white side of the isosurface and filled in red. The SD model shows a more connected morphology of each region, which becomes more strongly phase-separated than the TD model. In particular, the water-rich part in the SD model takes up less (or similar) space than that in the TD model, despite the high volume ratio of water beads in the SD model. Accordingly, these behaviors indicate that the monomer and polymer designs of the SD samples were appropriate for a high-performance PEM.

These results can be regarded as ironic from the point of view of conventional mesoscale simulation as well as PEM synthesis. Since the miscibility of materials depends on solubility parameters and their sizes, the ratio of hydrophilic beads with similar solubility parameters to water beads in a PEM model should be increased to obtain well-developed phase separation between the water/hydrophilic region and the hydrophobic region. This is also a well-accepted concept in synthesizing sulfonated polymers for PEM. However, in our results, although the TD model has a higher length of hydrophilic beads than the SD model, it shows weaker phase separation and water channel formation, despite the similar solubility values of T and S beads.

To understand this phenomenon, it is necessary to consider the water-channel formation mechanism in which water molecules are collected in the phase-separated hydrophilic region and thereby form the water channel. In a mesoscale simulation, this concept can be applied so that water beads are mixed with hydrophilic beads and then distributed through the hydrophilic region. Water beads start to aggregate and form a water channel. Accordingly, if the volumes of the hydrophilic region are the same, the model with a larger number of water beads will be advantageous for water channel formation. On the other hand, if the models have the same number of water beads, a smaller volume of the hydrophilic region will be advantageous for water channel formation. Thus, increasing the number of sulfonic acid groups per hydrophilic monomer increases IEC and water uptake. The SD model is more effective in phase separation and water channel formation than increasing the length of the hydrophilic block to increase IEC and water uptake.

**Figure 4:**
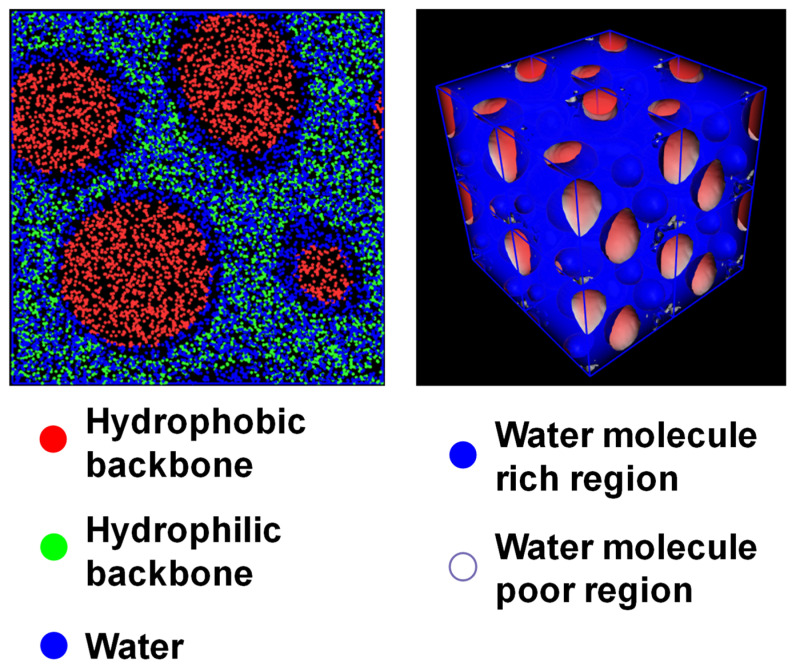
Sliced and 3D images of the mesoscale results of the modified SD model at 298 K after 10,000 steps.

This can be re-confirmed by the mesoscale simulation result, in which the length of the hydrophilic block in the SD model was doubled. As shown in Figure 4, although all of the remaining parameters are the same, the morphology of the modified SD model is entirely different from the original model. It is like that of the TD model. There is no water channel in the sliced image of the modified SD model, despite it having the same water volume ratio as the original model.

Based on the results discussed above, we can elucidate the well-defined water channel in Nafion membranes from a different point of view. In general, it is believed that the well-defined water channel in Nafion results from the high mobility of the side chain with sulfonic acid groups at the terminal end and the strong hydrophobicity of the PTFE backbone [10,12,48]. However, precisely speaking, this concept does not explain water channel formation but rather the phase separation between the hydrophilic moiety, including water molecules, and the hydrophobic moiety. Accordingly, it should be described why the water molecules aggregate effectively in the hydrophilic region of Nafion, which can be explained by the short length of its side chain compared to its backbone.

In addition, since the most significant difference in our 3D mesoscale models is the number of water beads defined from water uptake values, it is necessary to consider the effect of water molecules on the phase separation. When the density distribution evolves in the MesoDyn, the polymer model shows limited mobility due to the long chain structure. Still, miniature models, such as a solvent model consisting of one or two beads, can freely diffuse through the 3D mesoscale model. Accordingly, if two mesoscale models with bigger polymer and smaller solvent beads, have similar solubility parameters, the solvation effect can be expected to increase the mobility of the polymer models. In addition, the number of solvent beads should be enough to surround and separate the polymer beads. In the mesoscale PEM models of this study, water beads interact with hydrophilic beads due to their similar solubilities and can work as a solvent. As a result, the solvation effect of the water beads increases the mobility of the hydrophilic block, which helps to induce strong phase separation. Since the number of water beads in the SD model is higher than that in the TD model, it can be expected that the phase separation in the SD model will be faster and stronger. Of course, in actual fuel cell operations, excessive water uptake can reduce the processibility and/or durability of the PEMs. Accordingly, the degree of sulfonation should be carefully determined.

**Figure 5:**
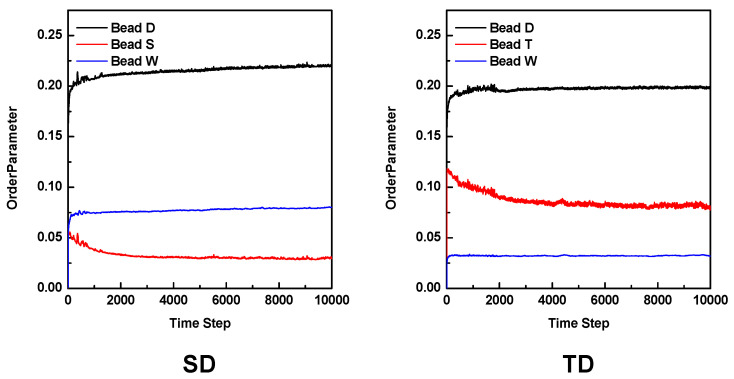
Order parameter as a function of the time step of the mesoscale models at 298 K after 10,000 steps.

Figure 5 shows the evolution of the order parameter as a function of the time step calculated from both mesoscale models. The order parameter is defined as follows [23,38]:

(9) $$P_{1}=\frac{1}{V}\int_{V} [\theta^{2}\left(r\right)-\theta_{A}{}^{2}]\mathrm{dr}$$

where $\theta_{I}\left(r\right)$ is a dimensionless density (volume fraction) for species A, since large and small values in the order parameters indicate stronger and weaker phase segregation, respectively. Figure 5 shows the change in the phase separation in our 3D mesoscale models according to time steps. In the case of the SD model, the order parameter of the hydrophilic S bead becomes stable, indicating well-equilibrated phase separation much faster than that of the hydrophilic T bead in the TD model, which can confirm our assumption discussed in the previous paragraph.

## 4. Conclusions

Using a mesoscale simulation, we successfullydemonstrated the differences in the phase preparation and water channel formation of sulfonated block copolymers synthesized for PEM applications. Two polyarylene sulfone-type polymers were selected as SD and TD models, in which the chemical structures of the hydrophobic block were the same but those of the hydrophilic block were different. In the SD model, the sulfonated monomer had a higher degree of sulfonation than in the TD model. Although the whole chemical structure of the SD and TD models was similar, the characteristics, such as water uptake, of the actual PEMs prepared using these polymer structures show significant differences. The SD model with sulfonated monomers, in which the number of sulfonic acid groups was twice that of the other model, showed better phase separation and water channel formation, despite the short length of its hydrophilic block. Considering the water-channel formation mechanism, this could be explained by the combined effects of higher water uptake and a shorter hydrophilic block in the SD model. For example, if the hydrophilic block lengths are the same, the larger water uptake model will be advantageous for water channel formation. On the other hand, if the models have the same water uptake, a shorter hydrophilic block will benefit water channel formation. In addition, due to the solvation effect of water beads, which increases the mobility of the hydrophilic block, the SD model had a larger number of water beads than the TD model, resulting in stronger and faster phase separation. Accordingly, these behaviors indicate that the monomer and polymer designs of the SD samples, affecting solubility parameter/molecular volume and topology, respectively, were appropriate as high-performance PEMs from the viewpoint of mesoscale simulation. This conclusion is supported by the experimental data, in which the proton conductivity of the SD PEM sample, 32.5 mS/cm, is higher than that of the TD PEM sample, 25.2 mS/cm [28], at 80 °C and in 50% RH conditions.

## Figures and Tables

**Table 1:** Characteristics of sulfonated polyarylene sulfone-multiblock copolymers used as PEM models in the mesoscale simulation of this study.

PEM Samples (hydrophilic M_n_/hydrophobic M_n_)	IEC_theoretical_ (meq/g)	Water Uptake (%)	Topology of PEM Model
TD (12 K/5 K)	1.88	35.18	T 17 D 12
SD (10 K/8 K)	2.47	77.52	S 12 D 19

**Table 2:** Solubility parameters and PEM model information for mesoscale simulation in this study.

PEM Model	Bead Name	Predicted by Synthia		Calculated by MD		Molecular Weight (g/mol)	Topology of PEM Model
		V_bead-mol_ (cm^3^/mol)	δ (MPa^1/2^)	V_bead-mol_ (cm^3^/mol)	δ (MPa^1/2^)		
TD	T	436.9	28.42	450.9	26.66	873	T 17 D 12
	D	318.9	22.69	327.2	19.62	419	
SD	S	238.9	27.84	244.1	26.69	410	S 12 D 19
	D	318.9	22.69	327.2	19.62	419	

## Data Availability

The data presented in this study are available on request from the corresponding author.
